# Extension of the reduced animal model to single-step methods

**DOI:** 10.1093/jas/skac272

**Published:** 2022-09-07

**Authors:** Mohammad Ali Nilforooshan

**Affiliations:** Livestock Improvement Corporation, Private Bag 3016, Hamilton 3240, New Zealand

**Keywords:** animal model, genotyped, parents, phenotyped, reduced model, single-step

## Abstract

For a few decades, animal models (AMs) in the form of best linear unbiased prediction (BLUP) have been used for the genetic evaluation of animals. An equation system is set in the order of all the effects in the model, including all the animals in the pedigree. Solving these large equation systems has been a challenge. Reduced AM (RAM) was introduced in 1980, which allowed setting up equations for parents instead of all animals. That greatly reduced the number of equations to be solved. The RAM is followed by a back-solving step, in which progenies’ breeding values are obtained conditional on parental breeding values. Initially, pedigree information was utilized to model genetic relationships between animals. With the availability of genomic information, genomic BLUP (GBLUP), single-step GBLUP (ssGBLUP), and single-step marker models were developed. Single-step methods utilize pedigree and genomic information for simultaneous genetic evaluation of genotyped and nongenotyped animals. There has been a shortage of studies on RAM development for genetic evaluation models utilizing genomic information. This study extended the concept of RAM from BLUP to the single-step methods. Using example data, three RAMs were described for ssGBLUP. The order of animal equations was reduced from the total number of animals to (1) genotyped animals and nongenotyped parents, (2) genotyped animals and nongenotyped phenotyped animals, and (3) genotyped animals and nongenotyped parents of phenotyped nongenotyped nonparents. Solutions for the remaining animals are obtained following a back-solving step. All the RAMs produced identical results to the full ssGBLUP. Advances in computational hardware have alleviated many computational limitations, but, on the other hand, the size of data is growing rapidly by the number of animals, traits, phenotypes, genotypes, and genotype density. There is an opportunity for a RAM comeback for the single-step methods to reduce the computational demands by reducing the number of equations.

## Teaser

Reduced animal models used to reduce computational demands for best linear unbiased prediction (BLUP) are now applicable to single-step genomic BLUP.

## Introduction

Since a few decades ago, animal genetic evaluations have been performed using “animal model” (AM) in the form of best linear unbiased prediction (BLUP; Henderson, 1975), taking into account genetic relationships between animals via pedigree information. With the availability of commercial genotyping platforms for livestock species, genomic BLUP (GBLUP; VanRaden, 2008) was developed, which was limited to information on genotyped animals. Followed by GBLUP, single-step methods were developed to incorporate pedigree and genomic information for simultaneous genetic evaluation of genotyped and nongenotyped animals. Single-step comes in the two forms of AM, called single-step GBLUP [ssGBLUP (Aguilar et al., 2010, Christensen and Lund, 2010)] and marker model (Fernando et al., 2014, 2016).

Worldwide, many genetic evaluation centers perform BLUP and single-step evaluations [ssGBLUP or single-step marker model (ssMM)] alongside each other, mainly for validation purposes and for qualifying their genomic data. Theoretically, genetic evaluation of animals unrelated to genotyped animals and their relatives remains intact in ssGBLUP compared with BLUP. However, minor differences are expected due to possibly different convergence behavior of BLUP and ssGBLUP equations (Vandenplas et al., 2018).

The concept of a reduced model (an equivalent to the full model) was first introduced to animal breeding by Henderson (1974), who absorbed equations for fixed effects into equations for sire effects. Quaas and Pollak (1980) took the reduced model to the next level by absorbing equations for random effects [reduced animal model (RAM)]. RAM allowed equations to be set up for parents in the mixed-model equations (MME). Since the number of parents is less than the number of progeny in most livestock populations, the order of equations to be formed and solved was greatly reduced (Mrode, 2005). This came with the huge benefit of reducing computational demands, given the computational limitations at the time. After solving RAM MME for breeding values of parents, breeding values of nonparents are obtained via back-solving predicted parental breeding values. The back-solving procedure is simpler and computationally less demanding than obtaining breeding values of nonparents via solving the MME for the full AM.

There have been tremendous advances in computational hardware, but on the other hand, data sizes used in genetic evaluations have increased considerably, mainly by larger pedigrees, more traits and phenotypes, and the incorporation of genomic data in genetic evaluations (VanRaden, 2008, Aguilar et al., 2010, Christensen and Lund, 2010, Fernando et al., 2014, 2016). There is an opportunity for RAM to reduce computational demands for modern (single-step) genetic evaluations.

Nilforooshan and Garrick (2021) introduced another RAM, which reduces the number of equations to phenotyped animals for BLUP, genotyped phenotyped animals for GBLUP, and genotyped animals and nongenotyped phenotyped animals for ssGBLUP. A back-solving step follows the RAM to calculate breeding values for animals not included in the MME. The aim of this technical note is to demonstrate the development of RAM for single-step methods, with numerical examples for ssGBLUP.

## Materials

The example data, code (written in the R programming language), and results of this study are publicly available (Nilforooshan, 2022).

### Ethical statement

No Animal Care and Use Committee approvals were required for this study because example data were used.

### Data

**Figure 1.**
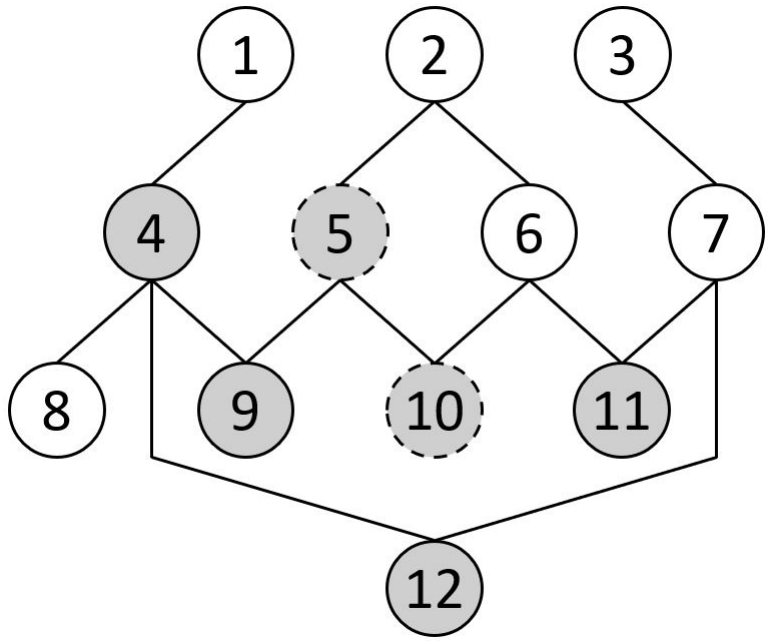
Pedigree structure. Genotyped animals are in dashed circles, and phenotyped animals are in a gray background.


The sample pedigree is illustrated in Figure 1, in which animals with odd numbers are females and animals with even numbers are males. Animals 5 and 10 are genotyped. Let us consider the genomic relationship matrix for these animals as

$$\begin{array}{l} G=\left[\begin{array}{ll} \;0.96\; & 0.56\;\\ \;0.56 & 1.16 \end{array}\right]. \end{array}$$

The phenotypes were 5.64, 4.30, 4.32, 5.39, 7.72, and 4.36 for animals 4, 5, 9, 10, 11, and 12, respectively. Genetic and residual variances were considered to be 1 and 2, respectively.

## Methods

### Pedigree pruning

Animals with no genetic information flow with genotyped animals might be discarded from the analysis. Breeding values of those animals are independent of genomic information and can be obtained via BLUP. Animal 3 in the example pedigree (Figure 1) is a removal candidate. Expressing the inverse of the pedigree-based additive genetic relationship matrix (**A**^−1^) as


$$\begin{array}{l} A^{-1}=\left[\begin{array}{llll} \;A^{00}\; & \;\;\;0 & \;\;\;0 & \;\;\;0\\ \;\;\;\;0 & A^{44}\; & A^{43} & \;\;\;0\\ \;\;\;\;0 & A^{34} & A^{33}\; & A^{32}\\ \;\;\;\;0 & \;\;\;0 & A^{23} & A^{22}\; \end{array}\right], \end{array}$$ (1)

where genotyped animals are denoted as 2, rows and columns corresponding to **A**^00^ can be discarded. **A**^33^ contains nongenotyped animals that are parent, or progeny, or mate to a genotyped animal, and **A**^44^ contains nongenotyped animals that are parent, or progeny, or mate to an animal in **A**^33^. Grouping


$$\begin{array}{l} \left[\begin{array}{ll} \;A^{44}\; & A^{43}\;\\ \;A^{34} & A^{33} \end{array}\right] \end{array}$$

into **A**^11^, **H**^−1^ (the inverse of the augmented pedigree- and genomic-based additive genetic relationship matrix) is reduced from


$$\begin{array}{l} \left[\begin{array}{lll} \;A^{00}\; & \;\;0 & \;\;0\\ \;\;0 & A^{11}\; & A^{12}\\ \;\;0 & A^{21} & H^{22}\; \end{array}\right]\;to\;\left[\begin{array}{ll} \;A^{11}\; & A^{12}\\ \;A^{21} & H^{22}\; \end{array}\right], \end{array}$$

where $H^{22}=A^{22}+G^{-1}-A_{22}^{-1},$$y^{\prime }=[{y^{\prime }}_{0}\;{y^{\prime }}_{1}\;{y^{\prime }}_{2}]$ is reduced to $[{y^{\prime }}_{1}\;{y^{\prime }}_{2}],$$X^{\prime }=\;\;\left[{X^{\prime }}_{0}\;{X^{\prime }}_{1}\;{X^{\prime }}_{2}\right]$ is reduced to $\left[{X^{\prime }}_{1}\;{X^{\prime }}_{2}\right]$, 


$$\begin{array}{l} Z=\left[\begin{array}{lll} \;Z_{0}\; & \;0 & \;\;0\\ \;\;0 & Z_{1}\; & \;0\\ \;\;0 & \;0 & Z_{2} \end{array}\right]\;is\;reduced\;to\;\left[\begin{array}{ll} \;Z_{1}\; & \;0\\ \;0 & Z_{2}\; \end{array}\right], \end{array}$$

and


$$\begin{array}{l} R^{-1}=\left[\begin{array}{lll} \;R^{00}\; & \;\;0 & \;\;0\\ \;\;0 & R^{11}\; & \;\;0\\ \;\;0 & \;\;0 & R^{22}\; \end{array}\right]\;is\;reduced\;to\;\left[\begin{array}{ll} \;R^{11}\; & \;\;0\\ \;\;0 & R^{22}\; \end{array}\right]. \end{array}$$

It is assuming no or negligible changes in the solution of fixed effects available in **X**_1_ and **X**_2_.

The ssGBLUP MME is written as


$$\begin{array}{l} \left[\begin{array}{ll} \;X^{\prime }R^{-1}X\; & \;X^{\prime }R^{-1}Z\\ \;Z^{\prime }R^{-1}X & Z^{\prime }R^{-1}Z+H^{-1}\sigma_{a}^{-2}\; \end{array}\right]\left[\begin{array}{l} \;\hat{b}\\ \;\hat{a}\; \end{array}\right]=\left[\begin{array}{l} \;X^{\prime }R^{-1}y\;\\ \;Z^{\prime }R^{-1}y \end{array}\right], \end{array}$$ (2)

where **y** is the vector of phenotypes, **X** is the incidence matrix relating **y** to fixed effects, **Z** is the incidence matrix relating **y** to animals, **R** is the diagonal matrix of residual variance(s) corresponding to **y**, $\sigma_{a}^{2}$ is the additive genetic variance, $\hat{b}$ is the vector of fixed effect solutions, and $\hat{a}$ is the vector of animals’ additive genetic merit solutions.

### Method 1

The RAM method of Quaas and Pollak (1980) for BLUP involves applying $y_{p}=X_{p}b+Z_{p}a_{p}+e_{p}$ for parents (*p*) and $y_{n}=X_{n}b+Z_{n}^{*}a_{p}+e_{n}^{*}$ for nonparents (*n*), where $Z_{n}^{*}$ is an incidence matrix of zeros and halves identifying the parents of animals (Mrode, 2005). This matrix has rows corresponding to phenotyped *n*, and columns corresponding to *p*. Considering


$$\begin{array}{l} \begin{array}{lll} & X= & \left[\begin{array}{l} X_{p}\\ X_{n} \end{array}\right],\\ & W= & \left[\begin{array}{l} Z_{p}\\ Z_{n}^{*} \end{array}\right],\\ & R^{-1}= & \left[\begin{array}{ll} R^{\;pp} & \;\;0\\ \;0 & \;R^{nn} \end{array}\right],\\ & R^{nn}= & {\left(R_{nn}+D_{nn}\sigma_{a}^{2}\right)}^{-1}, \end{array} \end{array}$$

and $D_{nn}$ being a diagonal block of the diagonal matrix **D**, where $A=TDT^{\prime }$, the RAM is written as


$$\begin{array}{l} \left[\begin{array}{ll} \;X^{\prime }R^{-1}X & \;X^{\prime }R^{-1}W\\ \;W^{\prime }R^{-1}X & \;W^{\prime }R^{-1}W+A_{pp}^{-1}\sigma_{a}^{-2} \end{array}\right]\left[\begin{array}{l} \hat{b}\\ {\hat{a}}_{p} \end{array}\right]=\left[\begin{array}{l} \;X^{\prime }R^{-1}y\\ \;W^{\prime }R^{-1}y\; \end{array}\right]. \end{array}$$ (3)

Compared with the full AM, **W**, $R^{-1}$, and $A_{pp}^{-1}$ replace


$$\begin{array}{l} Z=\left[\begin{array}{ll} \;Z_{p} & \;\;0\\ \;\;0 & \;Z_{n} \end{array}\right],\;R^{-1}=\left[\begin{array}{ll} \;R^{\;pp} & \;0\\ \;\;0 & \;R^{nn} \end{array}\right],\;and\;A^{-1}=\left[\begin{array}{ll} \;A^{\;pp} & \;A^{\;pn}\\ \;A^{np} & \;A^{nn}\; \end{array}\right], \end{array}$$

respectively. The difference between $R^{nn}$ and $R^{nn}$ is the absorption of the Mendelian Sampling term into the residual term (i.e., $e_{i}^{*}=MS_{i}+e_{i}$) for $R^{nn}$ (Mrode, 2005). Whereas, all animals are directly evaluated by AM (i.e., ${\hat{a}}^{\prime }=[{{\hat{a}}^{\prime }}_{p}\;{{\hat{a}}^{\prime }}_{n}]$), parents are directly evaluated by RAM (i.e., ${\hat{a}}_{p}$), and solutions for nonparents (${\hat{a}}_{n}$) are obtained indirectly via back-solving:


$$\begin{array}{l} \begin{array}{lll} & {\hat{a}}_{n}= & B(y_{n}-X_{n}\hat{b}-({\hat{a}}_{s}+{\hat{a}}_{d})/2)+({\hat{a}}_{s}+{\hat{a}}_{d})/2,\\ & B= & {\left(R^{nn}+D^{nn}\sigma_{a}^{-2}\right)}^{-1}R^{nn}, \end{array} \end{array}$$ (4)

where *s* and *d* are sire and dam of *n*. Matrix **B** can be simplified to ${\left(I+R_{nn}D^{nn}\sigma_{a}^{-2}\right)}^{-1}.$ To extend RAM to ssGBLUP, let us split animals into the three groups of genotyped animals (2), nongenotyped parents (*m*), and others (*n*). Combining *m* and 2 in *p*, Equation (2) can be written as


$$\begin{array}{ll} & \left[\begin{array}{lll} \;X^{\prime }R^{-1}X & \;{X^{\prime }}_{p}R^{\;pp}Z_{p} & \;X_{n}^{\prime }R^{nn}Z_{n}\\ \;{Z^{\prime }}_{p}R^{\;pp}X_{p} & {Z^{\prime }}_{p}R^{\;pp}Z_{p}+H^{\;pp}\sigma_{a}^{-2} & \;A^{\;pn}\sigma_{a}^{-2}\\ \;{Z^{\prime }}_{n}R^{nn}X_{n}\; & \;A^{np}\sigma_{a}^{-2} & \;{Z^{\prime }}_{n}R^{nn}Z_{n}+A^{nn}\sigma_{a}^{-2} \end{array}\right]\left[\begin{array}{l} \hat{b}\\ {\hat{a}}_{p}\\ {\hat{a}}_{n} \end{array}\;\right]\\ & =\left[\begin{array}{l} \;X^{\prime }R^{-1}y\\ {Z^{\prime }}_{p}R^{\;pp}y_{p}\\ {Z^{\prime }}_{n}R^{nn}y_{n} \end{array}\right], \end{array}$$ (5)

where


$$\begin{array}{l} \begin{array}{lll} & H^{\;pp}= & A^{\;pp}+\left[\begin{array}{ll} \;0 & \;0\\ \;0 & \;G^{-1}-A_{22}^{-1} \end{array}\right],\;A^{\;pp}=\left[\begin{array}{ll} \;A^{mm} & \;A^{m2}\;\\ \;A^{2m} & \;A^{22} \end{array}\right],\\ & {\hat{a}}_{p}= & \left[\begin{array}{l} {\hat{a}}_{m}\\ {\hat{a}}_{2} \end{array}\right],Z_{p}=\left[\begin{array}{ll} \;Z_{m} & \;\;0\\ \;0 & \;Z_{2} \end{array}\right],\;and\;R^{\;pp}=\left[\begin{array}{ll} \;R^{mm} & \;\;\;0\\ \;0 & \;R^{22}\; \end{array}\right]. \end{array} \end{array}$$

Following Quaas and Pollak (1980), Equation (5) is reduced to


$$\begin{array}{l} \left[\begin{array}{ll} X^{\prime }R^{-1}X & \;X^{\prime }R^{-1}W\\ W^{\prime }R^{-1}X & \;W^{\prime }R^{-1}W+H_{pp}^{-1}\sigma_{a}^{-2} \end{array}\right]\left[\begin{array}{l} \hat{b}\\ {\hat{a}}_{p} \end{array}\right]=\left[\begin{array}{l} X^{\prime }R^{-1}y\\ W^{\prime }R^{-1}y \end{array}\right], \end{array}$$ (6)

where


$$\begin{array}{l} H_{pp}^{-1}=A_{pp}^{-1}+\left[\begin{array}{ll} 0 & \;0\\ 0 & \;G^{-1}-A_{22}^{-1} \end{array}\right], \end{array}$$

and


$$\begin{array}{l} A_{pp}=\left[\begin{array}{ll} A_{mm} & \;A_{m2}\\ A_{2m} & \;A_{22} \end{array}\right]. \end{array}$$

Both $A_{22}^{-1}$ and $A_{pp}^{-1}$ are derived using the fast algorithm of Faux and Gengler (2013), which works based on pedigree searching for dependencies between selected animals or the method of Colleau (2002). Back-solving ${\hat{a}}_{n}$ is done using Equation (4). The reason for keeping nonparent genotyped animals in RAM is to keep the subsequent back-solving free from the blocks of $G^{-1}$. Otherwise, RAM would be computationally unjustifiable.

### Method 2

This method (Nilforooshan and Garrick, 2021) reduces AM for ssGBLUP to genotyped animals and nongenotyped phenotyped animals. Splitting animals to genotyped, nongenotyped phenotyped (*m*), and nongenotyped nonphenotyped (*n*), and combining *m* and 2 in *p*, Equation (2) can be written as


$$\begin{array}{ll} & \left[\begin{array}{lll} \;X^{\prime }R^{-1}X & \;{X^{\prime }}_{p}R^{\;pp}Z_{p} & \;X_{n}^{\prime }R^{nn}Z_{n}\\ \;{Z^{\prime }}_{p}R^{\;pp}X_{p} & {Z^{\prime }}_{p}R^{\;pp}Z_{p}+H^{\;pp}\sigma_{a}^{-2} & \;A^{\;pn}\sigma_{a}^{-2}\\ \;{Z^{\prime }}_{n}R^{nn}X_{n}\; & \;A^{np}\sigma_{a}^{-2} & \;{Z^{\prime }}_{n}R^{nn}Z_{n}+A^{nn}\sigma_{a}^{-2} \end{array}\right]\\ & \times \left[\begin{array}{l} \hat{b}\\ {\hat{a}}_{p}\\ {\hat{a}}_{n} \end{array}\;\right]=\left[\begin{array}{l} \;X^{\prime }R^{-1}y\\ {Z^{\prime }}_{p}R^{\;pp}y_{p}\\ {Z^{\prime }}_{n}R^{nn}y_{n} \end{array}\right], \end{array}$$ (7)

where $Z=[Z_{p}\;0],$ and matrix **0** corresponds to the columns of **Z** for animals *n*. The above equation can be reduced to


$$\begin{array}{l} \begin{array}{lll} & & \left[\begin{array}{ll} X^{\prime }R^{-1}X & \;X^{\prime }R^{-1}Z_{p}\\ {Z^{\prime }}_{p}R^{-1}X & \;{Z^{\prime }}_{p}R^{-1}Z_{p}+\Phi \sigma_{a}^{-2} \end{array}\right]\\ & & \;\times \left[\begin{array}{l} \hat{b}\\ {\hat{a}}_{p} \end{array}\right]=\left[\begin{array}{l} X^{\prime }R^{-1}y\\ {Z^{\prime }}_{p}R^{-1}y \end{array}\right], \end{array} \end{array}$$ (8)

where


$$\begin{array}{l} \begin{array}{lll} \Phi \; & =\;\; & H^{\;pp}-A^{\;pn}{\left(A^{nn}\right)}^{-1}A^{np}\\ & = & H^{\;pp}-A^{\;pp}+A_{pp}^{-1}\\ & = & A_{pp}^{-1}+\left[\begin{array}{l} 0\;0\\ 0\;G^{-1}-A_{22}^{-1} \end{array}\right]=H_{pp}^{-1}. \end{array} \end{array}$$

Back-solving for ${\hat{a}}_{n}$ solutions is done by solving


$$\begin{array}{l} A^{nn}{\hat{a}}_{n}=-A^{np}{\hat{a}}_{p}. \end{array}$$ (9)

### Method 3

This method further reduces the RAM created by Method 1. Consider the RAM presented in Equation (6). Dividing *p* (genotyped animals and nongenotyped parents) into genotyped animals and nongenotyped parents of phenotyped nongenotyped nonparents (*q*), and nongenotyped parents not parent to any phenotyped nongenotyped nonparent animal (*r*), Equation (6) can be written as


$$\begin{array}{l} \begin{array}{lll} & & \left[\begin{array}{lll} X^{\prime }R^{-1}X & \;X^{\prime }R^{-1}W_{q} & \;0\\ {W^{\prime }}_{q}R^{-1}X\; & {W^{\prime }}_{q}R^{-1}W_{q}+\Psi_{qq}\sigma_{a}^{-2} & \;\Psi_{qr}\sigma_{a}^{-2}\\ \;0 & \;\Psi_{rq}\sigma_{a}^{-2} & \;\Psi_{rr}\sigma_{a}^{-2} \end{array}\right]\\ & & \;\times \left[\begin{array}{l} \hat{b}\\ {\hat{a}}_{q}\\ {\hat{a}}_{r} \end{array}\right]=\left[\begin{array}{l} \;X^{\prime }R^{-1}y\\ {W^{\prime }}_{q}R^{-1}y\\ \;0 \end{array}\right], \end{array} \end{array}$$ (10)

where $W=[W_{q}\;0],$$W_{q}$ contains columns of **W** corresponding to *q*, and $\Psi =H_{pp}^{-1}.$ Next, the above equation system is reduced to


$$\begin{array}{l} \begin{array}{lll} & & \left[\;\begin{array}{ll} \;X^{\prime }R^{-1}X & \;X^{\prime }R^{-1}W_{q}\\ {W^{\prime }}_{q}R^{-1}X & \;{W^{\prime }}_{q}R^{-1}W_{q}+\Omega \sigma_{a}^{-2} \end{array}\;\right]\\ & & \;\times \left[\begin{array}{l} \hat{b}\\ {\hat{a}}_{q} \end{array}\right]=\left[\begin{array}{l} X^{\prime }R^{-1}y\\ {W^{\prime }}_{q}R^{-1}y \end{array}\right], \end{array} \end{array}$$ (11)

where $\Omega =(\Psi^{-1}{)}_{qq}^{-1}=(H_{pp}{)}_{qq}^{-1}=H_{qq}^{-1}.$ As such, the two-step reduction can be made at once with a one-step reduction to *q*. Back-solving ${\hat{a}}_{n}$ (solutions for nongenotyped nonparents) is done according to Equation (4), and back-solving for ${\hat{a}}_{r}$ solutions is done similar to Equation (9), by solving $A^{rr}{\hat{a}}_{r}=-A^{rq}{\hat{a}}_{q}.$

### Extension to the single-step marker model

Fernando et al. (2014) developed ssMM, an equivalent model to ssGBLUP, which uses imputed marker covariates for nongenotyped animals and a residual genetic effect accommodating deviations between imputed and true genotypes. Including an additive polygenic effect not captured by the markers (residual polygenic effect) into the marker model presented in Equation (3) of Fernando et al. (2016):


$$\begin{array}{l} \begin{array}{ll} & \left[\begin{array}{llll} \;X^{\prime }R^{-1}X & \;X^{\prime }R^{-1}ZM & \;X_{1}^{\prime }R^{11}Z_{1} & \;X^{\prime }R^{-1}Z\\ \;M^{\prime }Z^{\prime }R^{-1}X & \;M^{\prime }Z^{\prime }R^{-1}ZM+I\sigma_{\alpha }^{-2} & \;{M^{\prime }}_{1}{Z^{\prime }}_{1}R^{11}Z_{1} & \;M^{\prime }Z^{\prime }R^{-1}Z\\ \;{Z^{\prime }}_{1}R^{11}X_{1} & \;{Z^{\prime }}_{1}R^{11}Z_{1}M_{1} & \;{Z^{\prime }}_{1}R^{11}Z_{1}+A^{11}\sigma_{\alpha }^{-2} & \;\left[\begin{array}{ll} {Z^{\prime }}_{1}R^{11}Z_{1} & \;0 \end{array}\right]\\ \;Z^{\prime }R^{-1}X & \;Z^{\prime }R^{-1}ZM & \;{\left[\begin{array}{ll} {Z^{\prime }}_{1}R^{11}Z_{1} & \;0 \end{array}\right]}^{\prime } & \;Z^{\prime }R^{-1}Z+A^{-1}\sigma_{\delta }^{-2} \end{array}\right]\\ & \;\;\;\;\;\;\;\;\;\;\;\;\;\;\;\;\;\;\;\;\;\left[\begin{array}{l} \hat{b}\\ \hat{\alpha \;}\\ \hat{\epsilon }\\ \hat{\delta \;} \end{array}\right]=\left[\begin{array}{l} \;X^{\prime }R^{-1}y\\ M^{\prime }Z^{\prime }R^{-1}y\\ \;{Z^{\prime }}_{1}R^{11}y_{1}\\ \;\;Z^{\prime }R^{-1}y \end{array}\right] \end{array} \end{array}$$ (12)

where


$$\begin{array}{l} X=\left[\begin{array}{l} X_{1}\\ X_{2} \end{array}\right],\;y=\left[\begin{array}{l} y_{1}\\ y_{2} \end{array}\right],\;Z=\left[\begin{array}{ll} Z_{1} & \;\;0\\ \;0 & \;Z_{2} \end{array}\right],\;M=\left[\begin{array}{l} M_{1}\\ M_{2} \end{array}\right], \end{array}$$

**M**_2_ is the observed and centered genotype matrix for genotyped animals, **M**_1_ is the imputed genotype matrix for nongenotyped animals derived by solving **A**^11^**M**_1_ = −**A**^12^**M**_2_, $\sigma_{\alpha }^{2}$ is the additive genetic variance of marker effects, σδ2 is the residual polygenic variance, $\hat{\alpha }$ is the vector of marker effect solutions, $\hat{\epsilon }$ is the vector of predictions for the deviations between the true and estimated (imputed) marker breeding values for nongenotyped animals, and $\hat{\delta }$ is the vector of animal’s residual polygenic effect solutions.

#### Method 1

Applying Method 1 to Equation (12), the ssMM is reduced to genotyped animals and nongenotyped parents (*p*). Thus, **A**^−1^ is replaced with $A_{pp}^{-1},$**A**^11^ with ${\left(A_{pp}^{-1}\right)}_{\left(1\cap p)(1\cap p\right)}=A_{pp}^{\left(1\cap p)(1\cap p\right)},$**A**^12^ with ${\left(A_{pp}^{-1}\right)}_{(1\cap p)2}=A_{pp}^{(1\cap p)2},$$R^{nn}$ with $R^{nn}$, and $Z_{1}$ with $W_{1}$. Consequently, matrices made up of these matrices will change as well.

Back-solving for nongenotyped nonparents (*n*) is done by extending Equation (4) to


$$\begin{array}{l} \left[\begin{array}{l} {\hat{a}}_{n}\\ {\hat{\epsilon }}_{n} \end{array}\right]=\left[\begin{array}{ll} B & \;0\\ 0 & \;B \end{array}\right]\left[\begin{array}{l} y_{n}-X_{n}\hat{b}-({\hat{a}}_{s}+{\hat{a}}_{d})/2\\ y_{n}-X_{n}\hat{b}-({\hat{\epsilon }}_{s}+{\hat{\epsilon }}_{d})/2 \end{array}\right]+\frac{1}{2}\left[\begin{array}{l} {\hat{a}}_{s}+{\hat{a}}_{d}\\ {\hat{\epsilon }}_{s}+{\hat{\epsilon }}_{d} \end{array}\right]. \end{array}$$ (13)

If i∈n is not phenotyped, $y_{i}-X_{i}\hat{b}$ is replaced with $({\hat{a}}_{s}+{\hat{a}}_{d})/2.$ If the sire of i∈n is genotyped, ${\hat{\epsilon }}_{s_{i}}=0.$

#### Method 2

Applying Method 2 to Equation (12), the ssMM is reduced to genotyped animals and nongenotyped phenotyped animals (*p*). Thus, **A**^−1^ is replaced with $A_{pp}^{-1},$**A**^11^ with ${\left(A_{pp}^{-1}\right)}_{\left(1\cap p)(1\cap p\right)}=A_{pp}^{\left(1\cap p)(1\cap p\right)},$**A**^12^ with ${\left(A_{pp}^{-1}\right)}_{(1\cap p)2}=A_{pp}^{(1\cap p)2},$ and **Z**_1_ with $Z_{1\cap p}$. Consequently, matrices made up of these matrices will change too.

Back-solving for nongenotyped nonphenotyped animals (*n*) is performed by solving


$$\begin{array}{l} \left[\begin{array}{ll} A^{nn} & \;0\\ 0 & \;A^{nn} \end{array}\right]\left[\begin{array}{l} {\hat{a}}_{n}\\ {\hat{\epsilon }}_{n} \end{array}\right]=-\left[\begin{array}{ll} A^{np} & \;0\\ 0 & \;A^{np} \end{array}\right]\left[\begin{array}{l} {\hat{a}}_{p}\\ {\hat{\epsilon }}_{p} \end{array}\right]. \end{array}$$ (14)

#### Method 3

Applying Method 3 to Equation (12), the ssMM is reduced to genotyped animals and nongenotyped parents of ­phenotyped nongenotyped nonparents (*q*). Thus, **A**^−1^ is replaced with $A_{qq}^{-1},$**A**^11^ with ${\left(A_{qq}^{-1}\right)}_{\left(1\cap q)(1\cap q\right)}=A_{qq}^{\left(1\cap q)(1\cap q\right)},$**A**^12^ with ${\left(A_{qq}^{-1}\right)}_{(1\cap q)2}=A_{qq}^{(1\cap q)2},$$R^{nn}$ with $R^{nn},$ and $Z_{1}$ with $W_{1\cap q}.$ Consequently, matrices made up of these matrices will change too.

Back-solving for nongenotyped nonparents (*n*) is done according to Equation (13), and back-solving for nongenotyped parents, not parent to any phenotyped nongenotyped nonparent animal (*r*) is done by solving


$$\begin{array}{l} \left[\begin{array}{ll} A^{rr} & \;\;0\\ \;\;0 & \;A^{rr} \end{array}\right]\left[\begin{array}{l} {\hat{a}}_{r}\\ {\hat{\epsilon }}_{r} \end{array}\right]=-\left[\begin{array}{ll} A^{rq} & \;0\\ 0 & \;A^{rq} \end{array}\right]\left[\begin{array}{l} {\hat{a}}_{q}\\ {\hat{\epsilon }}_{q} \end{array}\right]. \end{array}$$

## Results

### Full animal model

The ssGBLUP MME for the full AM was formed and solved to check the correctness of the RAM solutions. Where applicable, columns/rows of matrices/vectors corresponding to animals are presented with animal identification indices. Where column and row indices are the same, only column indices are presented. The required matrices to form the MME were


$$\begin{array}{l} A^{-1}=\frac{1}{6}\begin{array}{l} \begin{array}{llllllllllll} \;1 & \;2 & \;3 & \;4 & \;5 & \;6 & \;7 & \;8 & \;9 & \;10 & \;11 & \;12 \end{array}\\ {}[\begin{array}{llllllllllll} \begin{array}{c} 8\\ 0\\ 0\\ -4\;\\ 0\\ 0\\ 0\\ 0\\ 0\\ 0\\ 0\\ 0 \end{array} & \begin{array}{c} 0\\ 10\\ 0\\ 0\\ -4\\ -4\\ 0\\ 0\\ 0\\ 0\\ 0\\ 0 \end{array} & \begin{array}{c} 0\\ 0\\ 8\\ 0\\ 0\\ 0\\ -4\;\\ 0\\ 0\\ 0\\ 0\\ 0 \end{array} & \begin{array}{c} -4\\ 0\\ 0\\ 16\\ 3\\ 0\\ 3\\ -4\\ -6\\ 0\\ 0\\ -6 \end{array} & \begin{array}{c} 0\\ -4\\ 0\\ 3\\ 14\\ 3\\ 0\\ 0\\ -6\\ -6\\ 0\\ 0 \end{array} & \begin{array}{c} 0\\ -4\\ 0\\ 0\\ 3\\ 14\\ 3\\ 0\\ 0\\ -6\\ -6\\ 0 \end{array} & \begin{array}{c} 0\\ 0\\ -4\\ 3\\ 0\\ 3\\ 14\\ 0\\ 0\\ 0\\ -6\\ -6 \end{array} & \begin{array}{c} 0\\ 0\\ 0\\ -4\\ 0\\ 0\\ 0\\ 8\\ 0\\ 0\\ 0\\ 0 \end{array} & \begin{array}{c} 0\\ 0\\ 0\\ -6\\ -6\\ 0\\ 0\\ 0\\ 12\\ 0\\ 0\\ 0 \end{array} & \begin{array}{c} 0\\ 0\\ 0\\ 0\\ -6\\ -6\\ 0\\ 0\\ 0\\ 12\\ 0\\ 0 \end{array} & \begin{array}{c} 0\\ 0\\ 0\\ 0\\ 0\\ -6\\ -6\\ 0\\ 0\\ 0\\ 12\\ 0 \end{array} & \begin{array}{c} 0\\ 0\\ 0\\ -6\\ 0\\ 0\\ -6\\ 0\\ 0\\ 0\\ 0\\ 12 \end{array}] \end{array} \end{array} \end{array}$$

$$\begin{array}{l} \begin{array}{lll} & H^{-1}= & A^{-1}+\left[\begin{array}{ll} 0 & \;0\\ 0 & \;G^{-1}-A_{22}^{-1} \end{array}\right],\\ & G^{-1}= & \begin{array}{ll} \;5 & \;10\\ {}[\begin{array}{l} 1.45\\ -0.7 \end{array} & \begin{array}{l} \;-0.7\\ \;1.2 \end{array}] \end{array},\\ & A_{22}^{-1}= & \begin{array}{ll} \;5 & \;10\\ {}[\begin{array}{l} 1.5319\\ -0.8511 \end{array} & \begin{array}{l} \;-0.8511\\ \;1.3617 \end{array}] \end{array},\\ & y{}^{\prime }\;= & \begin{array}{llllll} \begin{array}{l} \;4\\ {}[5.64 \end{array} & \begin{array}{l} \;5\\ \;4.30 \end{array} & \begin{array}{l} \;9\\ \;4.32 \end{array} & \begin{array}{l} \;10\\ \;5.39 \end{array} & \begin{array}{l} \;11\\ \;7.72 \end{array} & \begin{array}{l} \;12\\ \;4.36] \end{array} \end{array}, \end{array} \end{array}$$

$$\begin{array}{l} Z=\begin{array}{l} \begin{array}{llllllllllll} \;1 & \;2 & \;3 & \;4 & \;5 & \;6 & \;7 & \;8 & \;9 & \;\;10 & \;\;11 & \;\;12 \end{array}\\ \begin{array}{ll} \left[\begin{array}{llllllllllll} 0 & \;0 & \;0 & \;1 & \;0 & \;0 & \;0 & \;0 & \;0 & \;0 & \;0 & \;0\\ 0 & \;0 & \;0 & \;0 & \;1 & \;0 & \;0 & \;0 & \;0 & \;0 & \;0 & \;0\\ 0 & \;0 & \;0 & \;0 & \;0 & \;0 & \;0 & \;0 & \;1 & \;0 & \;0 & \;0\\ 0 & \;0 & \;0 & \;0 & \;0 & \;0 & \;0 & \;0 & \;0 & \;1 & \;0 & \;0\\ 0 & \;0 & \;0 & \;0 & \;0 & \;0 & \;0 & \;0 & \;0 & \;0 & \;1 & \;0\\ 0 & \;0 & \;0 & \;0 & \;0 & \;0 & \;0 & \;0 & \;0 & \;0 & \;0 & \;1 \end{array}\right] & \begin{array}{l} 4\\ 5\\ 9\\ 10\\ 11\\ 12 \end{array} \end{array} \end{array} \end{array}$$

$$\begin{array}{l} X^{\prime }=\begin{array}{ll} \begin{array}{llllll} \;4 & \;5 & \;9 & \;10 & \;11 & \;12 \end{array} & \\ \left[\begin{array}{llllll} 1 & \;1 & \;1 & \;1 & \;\;1 & \;\;1\\ 0 & \;1 & \;1 & \;0 & \;\;1 & \;\;0 \end{array}\right] & \begin{array}{l} \mu \\ sex \end{array} \end{array}, \end{array}$$

$$\begin{array}{l} R^{-1}=I_{6}\sigma_{e}^{-2}, \end{array}$$

where $\sigma_{e}^{2}$ is the residual variance. Solving Equation (2) returns


$$\begin{array}{l} \hat{b}=\left[\begin{array}{l} 5.1938\\ 0.3287 \end{array}\right],\;\hat{a}=\left[\begin{array}{l} -0.0476\\ -0.0414\\ 0.1040\\ -0.0953\\ -0.4302\\ 0.3268\\ 0.2080\\ -0.0476\\ -0.4507\\ 0.0255\\ 0.6534\\ -0.1217 \end{array}\right]\begin{array}{l} 1\\ 2\\ 3\\ 4\\ 5\\ 6\\ 7\\ 8\\ 9\\ 10\\ 11\\ 12 \end{array}. \end{array}$$

### RAM: Method 1

Reducing AM to genotyped animals and nongenotyped parents (p={1−7,  10}), nongenotyped nonparents (*n* = {8, 9, 11, 12}) are excluded from the model. Changing from AM to RAM, $R^{-1}$ replaces $R^{-1},$**W** replaces **Z**, and $H_{pp}^{-1}$ replaces **H**^−1^, where


$$\begin{array}{l} D_{nn}=\begin{array}{l} \begin{array}{lll} \;\;\;\;9 & \;\;\;11 & \;\;\;12 \end{array}\\ diag(\begin{array}{lll} 0.5 & \;0.5 & \;0.5 \end{array}) \end{array} \end{array}$$

$$\begin{array}{l} R^{nn}=\begin{array}{l} \begin{array}{lll} \;\;\;9 & \;11 & \;12 \end{array}\\ diag(\begin{array}{lll} 0.4 & \;0.4 & \;0.4 \end{array}) \end{array} \end{array}$$

$$\begin{array}{l} W=\begin{array}{ll} \begin{array}{llllllll} \;1 & \;2 & \;3 & \;4 & \;5 & \;6 & \;7 & \;10 \end{array} & \\ \left[\begin{array}{llllllll} 0 & \;0 & \;0 & \;1 & \;0 & \;0 & \;0 & \;0\\ 0 & \;0 & \;0 & \;0 & \;1 & \;0 & \;0 & \;0\\ 0 & \;0 & \;0 & \;0.5 & \;0.5 & \;0 & \;0 & \;0\\ 0 & \;0 & \;0 & \;0 & \;0 & \;0 & \;0 & \;1\\ 0 & \;0 & \;0 & \;0 & \;0 & \;0.5 & \;0.5 & \;0\\ 0 & \;0 & \;0 & \;0.5 & \;0 & \;0 & \;0.5 & \;0 \end{array}\right] & \begin{array}{l} 4\\ 5\\ 9\\ 10\\ 11\\ 12 \end{array} \end{array}, \end{array}$$

$$\begin{array}{l} A_{pp}^{-1}=\frac{1}{6}\begin{array}{l} \begin{array}{cccccccc} \;1 & \;2 & \;3 & \;4 & \;5 & \;6 & \;7 & \;10 \end{array}\\ \left[\begin{array}{cccccccc} 8 & 0 & 0 & -4 & 0 & 0 & 0 & 0\\ 0 & 10 & 0 & 0 & -4 & -4 & 0 & 0\\ 0 & 0 & 8 & 0 & 0 & 0 & -4 & 0\\ -4 & 0 & 0 & 8 & 0 & 0 & 0 & 0\\ 0 & -4 & 0 & 0 & 11 & 3 & 0 & -6\\ 0 & -4 & 0 & 0 & 3 & 11 & 0 & -6\\ 0 & 0 & -4 & 0 & 0 & 0 & 8 & 0\\ 0 & 0 & 0 & 0 & -6 & -6 & 0 & 12 \end{array}\right] \end{array}, \end{array}$$

$$\begin{array}{l} H_{pp}^{-1}=A_{pp}^{-1}+\left[\begin{array}{ll} 0 & \;0\\ 0 & \;G^{-1}-A_{22}^{-1} \end{array}\right]. \end{array}$$

Note that even though 8∈n, because it has no phenotype, it has no designated rows and columns in **D**_*nn*_, $R^{nn}$, and rows in **W**. Solving Equation (6) returns


$$\begin{array}{l} \hat{b}=\left[\begin{array}{l} 5.1938\\ 0.3287 \end{array}\right],\;{\hat{a}}_{p}=\left[\begin{array}{l} -0.0476\\ -0.0414\\ 0.1040\\ -0.0953\\ -0.4302\\ 0.3268\\ 0.2080\\ 0.0255 \end{array}\right]\begin{array}{l} 1\\ 2\\ 3\\ 4\\ 5\\ 6\\ 7\\ 10 \end{array} \end{array}$$

Back-solving involves Equation (4), where


$$\text{B}=\frac{1}{11}\text{diag}\begin{array}{c} \begin{array}{cccc} 8 & 9 & 11 & 12 \end{array}\\ \left(\begin{array}{cccc} 3 & 2.2 & 2.2 & 2.2 \end{array}\right) \end{array}$$

$$\begin{array}{l} (y_{n}-X_{n}\hat{b}{)}^{\prime }=\begin{array}{llll} \begin{array}{l} \;8\\ {}[({\hat{a}}_{s_{8}}+{\hat{a}}_{d_{8}})/2 \end{array} & \begin{array}{l} \;9\\ \;-\;1.2025 \end{array} & \begin{array}{l} \;11\\ \;2.1975 \end{array} & \begin{array}{l} \;12\\ \;-0.8338] \end{array} \end{array}, \end{array}$$

$$\begin{array}{l} {{\hat{a}}^{\prime }}_{s}=\begin{array}{llll} \begin{array}{l} \;8\\ {}[-0.0953 \end{array} & \begin{array}{l} \;9\\ \;-0.0953 \end{array} & \begin{array}{l} \;11\\ \;0.3268 \end{array} & \begin{array}{l} \;12\\ \;-0.0953] \end{array} \end{array}, \end{array}$$

$$\begin{array}{l} {{\hat{a}}^{\prime }}_{d}=\begin{array}{llll} \begin{array}{l} \;8\\ {}[0 \end{array} & \begin{array}{l} \;9\\ \;-\;0.4302 \end{array} & \begin{array}{l} \;11\\ \;0.2080 \end{array} & \begin{array}{l} \;12\\ \;0.2080] \end{array} \end{array}, \end{array}$$

and returns


$$\begin{array}{l} {{\hat{a}}^{\prime }}_{n}=\begin{array}{llll} \begin{array}{l} \;8\\ {}[-0.0476 \end{array} & \begin{array}{l} \;9\\ \;-0.4507 \end{array} & \begin{array}{l} \;11\\ \;0.6534 \end{array} & \begin{array}{l} \;12\\ \;-0.1217] \end{array} \end{array}. \end{array}$$

Notice that for nonphenotyped animal i∈n,$({\hat{a}}_{s_{i}}+{\hat{a}}_{d_{i}})/2$ is assigned to $y_{i}-X_{i}\hat{b}.$

### RAM: Method 2

Reducing AM to genotyped animals and nongenotyped phenotyped animals (p={4, 5, 9−12}), nongenotyped nonphenotyped animals (*n* = {1, 2, 3, 6, 7, 8}) are excluded from the model. Changing from AM to RAM, **Z**_*p*_ replaces **Z**, and $H_{pp}^{-1}$ replaces **H**^−1^, where


$$\begin{array}{l} Z_{p}=\begin{array}{l} \begin{array}{llllllll} & \;4 & \;5 & \;9 & \;10 & \;11 & \;12 & \end{array}\\ \left[\begin{array}{llllll} 1 & \;0 & \;0 & \;0 & \;\;0 & \;\;0\\ 0 & \;1 & \;0 & \;0 & \;\;0 & \;\;0\\ 0 & \;0 & \;1 & \;0 & \;\;\;0 & \;\;0\\ 0 & \;0 & \;0 & \;1 & \;\;0 & \;\;0\\ 0 & \;0 & \;0 & \;0 & \;\;1 & \;\;0\\ 0 & \;0 & \;0 & \;0 & \;\;0 & \;\;1 \end{array}\right] \end{array}, \end{array}$$

$$\begin{array}{l} A_{pp}^{-1}=\begin{array}{l} \begin{array}{llllllllllllllllll} & & \;4 & & & \;5 & & & \;9 & & \;10 & & \;11 & & & \;12 & & \end{array}\\ \left[\begin{array}{cccccc} 1.8670 & 0.5150 & -1 & -0.0644 & 0.2017 & -0.7339\\ 0.5150 & 2.0386 & -1 & -0.8798 & 0.0901 & -0.0300\\ -1 & -1 & 2 & 0 & 0 & 0\\ -0.0644 & -0.8798 & 0 & 1.4850 & -0.3863 & 0.1288\\ 0.2017 & 0.0901 & 0 & -0.3863 & 1.2103 & -0.4034\\ -0.7339 & -0.0300 & 0 & 0.1288 & -0.4034 & 1.4678 \end{array}\right] \end{array}, \end{array}$$

$$\begin{array}{l} H_{pp}^{-1}=A_{pp}^{-1}+\left[\begin{array}{ll} 0 & \;0\\ 0 & \;G^{-1}-A_{22}^{-1} \end{array}\right]. \end{array}$$

Solving Equation (8) returns


$$\begin{array}{l} \hat{b}=\left[\begin{array}{l} 5.1938\\ 0.3287 \end{array}\right],\;{\hat{a}}_{p}=\left[\begin{array}{l} -0.0953\\ -0.4302\\ -0.4507\\ 0.0255\\ 0.6534\\ -0.1217 \end{array}\right]\begin{array}{l} 4\\ 5\\ 9\\ 10\\ 11\\ 12 \end{array}. \end{array}$$

Back-solving involves Equation (9), and returns


$$\begin{array}{l} {{\hat{a}}^{\prime }}_{n}=\begin{array}{llllll} \;1 & \;2 & \;3 & \;6 & \;7 & \;8\\ {}[-0.0476 & \;-0.0414 & \;0.1040 & \;0.3268 & \;0.2080 & \;-0.0476] \end{array}. \end{array}$$

### RAM: Method 3

Reducing AM to genotyped animals and nongenotyped parents of phenotyped nongenotyped nonparents (q={4−7,10}), nongenotyped nonparents (n={8,9,11,12}) and nongenotyped parents not parent to any phenotyped nongenotyped nonparent animal (*r* = {1, 2, 3}) are excluded from the model. Changing from AM to RAM, $R^{-1}$ replaces $R^{-1}$, $W_{q}$ replaces **Z**, and $H_{qq}^{-1}$ replaces $H^{-1},$ where


$$\begin{array}{l} Wq=\begin{array}{l} \begin{array}{lllll} \;4 & \;5 & \;6 & \;7 & \;10 \end{array}\\ \left[\begin{array}{lllll} \;1 & \;0 & \;0 & \;0 & \;0\\ \;0 & \;1 & \;0 & \;0 & \;0\\ 0.5 & \;0.5 & \;0 & \;0 & \;0\\ \;0 & \;0 & \;0 & \;0 & \;1\\ \;0 & \;0 & \;0.5 & \;0.5 & \;0\\ 0.5 & \;\;\;0 & \;0 & \;0.5 & \;0 \end{array}\right]\begin{array}{l} 4\\ 5\\ 9\\ 10\\ 11\\ 12 \end{array} \end{array}, \end{array}$$

$$\begin{array}{l} A_{qq}^{-1}=\frac{1}{3}\begin{array}{l} \begin{array}{llllllll} & \;\;4 & \;\;5 & & \;\;6 & \;\;7 & \;10 & \end{array}\\ \left[\begin{array}{lllll} 3 & \;0 & \;0 & \;0 & \;0\\ 0 & \;4.7 & \;0.7 & \;0 & \;-\;\;3\\ 0 & \;0.7 & \;4.7 & \;0 & \;-\;\;3\\ 0 & \;0 & \;0 & \;3 & \;0\\ 0 & \;-\;\;3 & \;-\;\;3 & \;0 & \;6 \end{array}\right] \end{array}, \end{array}$$

$$\begin{array}{l} H_{qq}^{-1}=A_{qq}^{-1}+\left[\begin{array}{ll} 0 & \;0\\ 0 & \;G^{-1}-A_{22}^{-1} \end{array}\right]. \end{array}$$

Solving Equation (11) returns


$$\begin{array}{l} \begin{array}{lll} & {\hat{b}}^{\prime }= & \left[\begin{array}{ll} 5.1938 & 0.3287 \end{array}\right],\\ & {{\hat{a}}^{\prime }}_{q}= & \begin{array}{lllll} \begin{array}{l} 4\\ {}[-0.0953 \end{array} & \begin{array}{l} 5\\ -0.4302 \end{array} & \begin{array}{l} 6\\ -0.3268 \end{array} & \begin{array}{l} 7\\ 0.2080 \end{array} & \begin{array}{l} 10\\ 0.0255] \end{array} \end{array}. \end{array} \end{array}$$

Back-solving for ${\hat{a}}_{n}$ solutions involves Equation (4), and returns


$$\begin{array}{l} {{\hat{a}}^{\prime }}_{n}=\begin{array}{llll} \begin{array}{l} 8\\ {}[-0.0476 \end{array} & \begin{array}{l} 9\\ -0.4507 \end{array} & \begin{array}{l} 11\\ 0.6534 \end{array} & \begin{array}{l} 12\\ -0.1217] \end{array} \end{array}. \end{array}$$

Back-solving for ${\hat{a}}_{r}$ solutions involves solving $A^{rr}{\hat{a}}_{r}=-A^{rq}{\hat{a}}_{q}$, and returns


$$\begin{array}{l} {{\hat{a}}^{\prime }}_{r}=\begin{array}{lll} \begin{array}{l} 1\\ {}[-0.0476 \end{array} & \begin{array}{l} 2\\ -0.0414 \end{array} & \begin{array}{l} 3\\ 0.1040] \end{array} \end{array}. \end{array}$$

#### Factors affecting the computational efficiency of RAM: A real data example

The computational efficiency of RAM depends on the size and the computational cost of deriving $A_{qq}^{-1}$ vs. $A^{-1}$ , and the sparsity of the rows of $A_{qq}^{-1}$ corresponding to nongenotyped animals (the block of $A_{qq}^{-1}$ corresponding to genotyped animals sums with $G^{-1}-A_{22}^{-1},$ which is dense). With optimized computing, the computational costs of forming $W_{q}$ and $R^{-1}$ are marginally greater than those for **Z** and $R^{-1}$ for the full AM. This is considering that the back-solving procedure should remain as simple and efficient as possible. The most important factors influencing (some with overlapping influence) the computational efficiency of the ssGBLUP RAM are (1) number of genotyped vs. nongenotyped animals, (2) number of nongenotyped parents vs. nongenotyped nonparents, (3) number of nongenotyped phenotyped vs. nongenotyped nonphenotyped animals, (4) pedigree missing rate, and (5) sparsity of the rows of $A_{qq}^{-1}$ corresponding to nongenotyped animals (influenced by the sparsity of $A^{-1}$).

A real data (New Zealand’s dairy cattle pedigree, May 2022) example was used to study the dimension reduction by RAM. There were 32,679,354 animals in the pedigree. Considering Equation (1), there were 243,034, 405,911, 25,759,086, and 6,335,004 animals in **A**^22^, **A**^33^, **A**^44^, and **A**^00^, respectively. Pruning the pedigree free from animals in **A**^00^, 26,344,350 animals remained in the pedigree. There were 13,980,955 nongenotyped parents in the remaining pedigree, of which 9,041,130 were parents of nongenotyped nonparents. The 243,034 genotyped animals and the 9,041,130 nongenotyped parents of nongenotyped nonparents remained for the single-step RAM. Without pedigree pruning, 243,034 genotyped animals and 10,362,206 nongenotyped parents of nongenotyped nonparents remained in the RAM. Since the aim is to keep the back-solving procedure free from genomic information, only the equations corresponding to nongenotyped animals undergo reduction. It was possible to further reduce nongenotyped parents of nongenotyped nonparents to nongenotyped parents of phenotyped nongenotyped nonparents. However, this further reduction was omitted, so that a single $A_{qq}^{-1}$ is created and used in all the analyses for different traits. Similarly, the ssMM equations (Equation (12)) corresponding to nongenotyped animals are reduced to nongenotyped parents of nongenotyped nonparents.

## Discussion

Breeding values of some nongenotyped animals are not influenced and do not influence the breeding values of genotyped animals. If BLUP is performed in conjunction with single-step evaluations, those animals can be evaluated via BLUP only.

This study showed that RAM used to reduce the dimension of equations for BLUP can be easily extended to single-step genetic evaluations. Numerical results were shown for ssGBLUP, and the three RAMs produced identical solutions to the full ssGBLUP. No results were presented for the ssMM. However, as the ssMM is an equivalent form of ssGBLUP (Fernando et al., 2014, Bermann et al., 2022), indirectly evaluating animals via directly evaluating marker effects, the same principles applied to RAM for ssGBLUP are valid for the reduced ssMM. Considering Equation (12) and Method 3, the number of equations corresponding to $\hat{\epsilon }$ reduces from the number of nongenotyped animals to nongenotyped parents of phenotyped nongenotyped nonparents, and the number of equations corresponding to $\hat{\delta }$ reduces from the total number of animals to genotyped animals and nongenotyped parents of phenotyped nongenotyped nonparents.

Compared with RAM for BLUP, genotyped animals are present in the reduced single-step model. An efficient reduced model should be followed by an easy and fast back-solving, introducing no new information other than the conditionality of the back-solving solutions to the RAM solutions. That conditionality should be defined via sparse matrices, such as the diagonal matrix **B** in Methods 1 and Method 3, or blocks of **A**^−1^ in Methods 2 and 3, instead of the dense blocks of **H**^−1^.

The choice among the three RAMs depends on the number of nongenotyped animals remaining together with genotyped animals in RAM. It seems that Method 2 compromises the sparseness of $A_{pp}^{-1}$ used in $H_{pp}^{-1},$ and that back-solving ${\hat{a}}_{n}$ is easier in Methods 1 and 3. Therefore, unless the number of nongenotyped phenotyped animals is considerably less than the number of nongenotyped parents or nongenotyped parents of phenotyped nongenotyped nonparents, Methods 1 and 3 are preferred over Method 2.

## Abbreviations

AM: animal model
BLUP: best linear unbiased prediction
GBLUP: genomic BLUP
MME: mixed-model equations
RAM: reduced AM
ssGBLUP: single-step GBLUP
ssMM: single-step marker model
